# Wardleworth, on Black Pitch in the Treatment of Hæmorrhoids

**Published:** 1842-10-01

**Authors:** T. H. Wardleworth


*Black Pitch in the Treatment of Hæmorrhoids.* By T. H. Wardleworth, M. D.—Hæmorrhoids, anatomically speaking, are hypertrophied cellular tissue, in connection with a large number of varicose veins ; they are of an indolent or inflammatory character, generally appearing in those persons who live well, and take but little exercise. Out of eighty cases that have come under my notice, sixty occurred in males, and twenty in females; their origin in the latter could be traced to the free use of the pills of the Whitworth doctor, of bone-setting notoriety. These pills are chiefly composed of aloes ; in the former the piles were produced in twenty from hard riding ; in fifteen their occurrence was attributed to the circumstance of having sat some time upon wet grass ; and in the remaining twenty-five, a variety of causes were assigned for the production of hæmorrhoids. The most troublesome symptoms in those patients whose piles resulted from hard riding, were a continual itching about the anus, accompanied with a burning sensation ; and to use the expression of the patients themselves, the heat was so intense that it seemed as if " a red-hot iron had been thrust up the fundament." Upon an examination of these persons, a considerable degree of fulness around the verge of the anus was discovered, attended with an inflammatory blush of a leaden hue ; the internal surface of the rectum presented to the finger a considerable number of prominences about the size of horse-beans, situated just within the sphincter ani, which were exceedingly tender to the touch, for on gently pressing them with the point of the finger, the patient complained of a severe lancinating pain ; considerable annoyance was experienced from protrusion of the piles after each motion, but no hæmorhage followed.

The treatment consisted in the application of leeches to the perineum, and in the neighbourhood of the coccyx ; for I have witnessed very unpleasant effects from placing leeches upon the verge of the anus when much swollen and inflamed, and more particularly when the inflamed surface has had a dark or leaden appearance ; in such cases I have seen small ulcers produced by the leech bites that have required more care and attention to heal than the removal of the piles, and, in one instance, considerable sloughing was the result. I am happy to find that my experience perfectly accords with that of M. Lisfranc, as far as regards the application of leeches to the inflamed parts ; and I think with him that the strictest care is necessary in the employment of leeches, and that they ought never to be applied to the anus when in a state of inflammation. After the inflammatory symptoms have subsided, I administered common black pitch, and under its use a speedy cure was affected. In the remaining sixty patients the piles were of an indolent character ; in ten, they were attended with a most troublesome discharge of mucus and blood, both before and after a motion. In the remaining fifty, the piles were external; and in thirty instances they had existed for more than six years, and during that time almost every kind of treatment had been adopted, but without benefit. In one or two of the patients there were evident symptoms of disease of the liver, but in the remainder it seemed that the piles were unaccompanied with any visceral disturbances or organic disease.

Having witnessed the good effects of black pitch in the treatment of piles of an indolent kind, whether attended with hæmorrhage or not, I at once commence with the administration of that substance, combining it with a little blue pill in the two cases already referred to, in whom I apprehended disease of the liver. When given agreeably to the following formula:—Take of black pitch, one drachm; powdered gum arabic, half a drachm; well mixed together, and divided into twenty pills, two of which are to be taken every night; its action upon the bowels is that of a gentle aperient; thereby rendering the pitch a most desirable remedy in piles. As a remedial agent in affections of an indolent character situated in the vicinity of the rectum, black pitch has, according to my experience, a decided superiority over *Ward's paste*, although the latter has been introduced into the pharmacopœia. *Ward's paste*, as just observed, is far inferior to black pitch as a medicinal remedy in relieving those distressing affections of the rectum, fistula in ano, and hæmorrhoids; for, on comparing the number of cases of fistula treated with *Ward's paste*, and black pitch, the latter had the advantage both as regards the number of cures and the time in which they were accomplished. With such an agent as this, in the affections referred to, I do think that the profession ought rather to employ it than to encourage the use of a quack medicine. Much has been said in your Journal upon the subject of medical reform; and certainly reform in this particular is of the first importance, inasmuch as the sale of quack medicines has always and ever will tend to injure the medical practitioner.

But in returning to the subject of the paper, I may state my opinion, which is based upon considerable experience, that neither the ligature nor the knife will ever be wanted (except in extreme cases which have been neglected) in the cure of piles, or of fistula in ano. "The true end of surgery is to preserve, not to destroy," says M. Lisfranc. Bateman recommends pitch in several cutaneous affections as a powerful stimulant, and as possessing the property of improving that acid condition of the skin which generally accompanies cutaneous affections. Supposing, *à priori*, that pitch acts thus beneficially upon the functions of the skin, we may fairly infer that by gently stimulating the capillaries and exhalents on the surface of the intestinal canal, it may produce a healthy action, so necessary to the relief of the dilated hæmorrhoidal vessels and hypertrophied cellular tissue. In this way the influence of the pitch may be exercised upon the portal veins, causing an acceleration of this portion of the venous circulation, and together with its action upon the bowels, it may also probably increase the biliary circulation. A chain of effects occurring like what I have attempted but feebly to explain, would, upon true physiological principles, remove the sluggish state of the circulation which was present in sixty out of the eighty cases as treated by me. Instances will occur in which the pitch would be inadmissible, viz., pregnancy, and in a highly irritable condition of the bowels, unless combined with opium. With these exceptions pitch is the only remedy, in conjunction with frequent injections of tepid water into the rectum, being satisfied that cleanliness is of the first importance in the treatment of diseases in that vicinity, on which I rely for the removal of piles. In fistula, its use has been attended with the best results; therefore I can recommend the pitch to the notice of my professional brethren.—*London Lancet*, August 27, 1842.

